# Patient perception of bowel urgency and remission in moderately to severely active Crohn’s disease or ulcerative colitis: a qualitative study

**DOI:** 10.1186/s41687-024-00800-1

**Published:** 2024-11-12

**Authors:** Theresa Hunter Gibble, Larissa Stassek, Gale Harding, Marissa Stefan, Tsion Fikre, Alison Potts Bleakman, Richard Moses, Marla Dubinsky

**Affiliations:** 1 Eli Lilly and Company, 893 S Delaware St., Indianapolis, IN 46225 USA; 2 Evidera, Bethesda, MD USA; 3 Icahn School of Medicine at Mount Sinai, New York, NY USA

**Keywords:** Crohn’s disease, Ulcerative colitis, Bowel urgency, Qualitative interview, Patient-reported outcome, Urgency Numeric Rating Scale

## Abstract

**Background:**

Bowel urgency, the sudden and immediate need to have a bowel movement, is common in patients with ulcerative colitis (UC) and Crohn’s disease (CD). While its impact in UC is well established, less is known about its importance in CD. Further, what level of bowel urgency control patients with UC or CD would consider to be acceptable or in remission has not been established. This qualitative study aimed to understand perceptions of bowel urgency and remission in these patients.

**Methods:**

Semi-structured combined concept elicitation and cognitive interviews were conducted to explore how adults with moderate-to-severe UC or CD and current or recent bowel urgency think about the concept of bowel urgency and its remission. The Urgency Numeric Rating Scale (UNRS) was used to examine different levels of bowel urgency severity and to investigate what level of bowel urgency patients would consider as representing remission.

**Results:**

Forty adults (*n* = 19 UC, *n* = 21 CD) recruited from six US sites completed the study. Sociodemographic and clinical characteristics were similar in both groups. Both groups reported impacts of bowel urgency on physical, social, professional, and emotional aspects of their lives. Most participants (*n* = 15 UC, *n* = 18 CD) reported having experienced one or more incidents of urgency-related fecal incontinence. Most participants considered remission to be a state with a normal or reduced number of bowel movements and no or less frequent bowel urgency, and they noted that remission would increase their ability to engage in daily activities without fear of fecal incontinence. Participants were able to map different levels of severity of bowel urgency to UNRS score ranges (scale: 0–10), and they indicated that a mean UNRS score of 5 (UC) or 4 (CD) would be the highest point on the NRS at which they would still consider their bowel urgency to be in remission.

**Conclusions:**

Bowel urgency is an important issue for patients with either moderate-to-severe UC or CD, and its remission would improve their lives. Further, these patients may still consider bowel urgency to be in remission even at UNRS scores as high as 4 or 5.

## Abstract (plain language summary)

Bowel urgency is the sudden need to rush to the bathroom to have a bowel movement, which is common in people with ulcerative colitis and Crohn’s disease. We interviewed adults with moderate or severe ulcerative colitis or Crohn’s disease to better understand their experiences. They said that bowel urgency often made them feel anxious, depressed, or embarrassed and interfered with their social lives, work, and chores. Most felt that they would be in “remission” if the bowel urgency stopped or happened less often. They added that this would help them feel more relaxed and be more active. Interestingly, they would consider even a moderate amount of bowel urgency as being in “remission.” This information can help healthcare providers better understand the toll that bowel urgency takes on patients with ulcerative colitis or Crohn’s disease and better manage their treatment.

## Background

Crohn’s disease (CD) and ulcerative colitis (UC) are chronic, relapsing forms of inflammatory bowel disease [1–3]. UC is characterized by mucosal inflammation of the colon and rectum, and common symptoms include blood in the stool, diarrhea, and abdominal pain [3, 4]. In contrast to UC, CD can affect any part of the digestive system, including the large and small bowel, perianal region, stomach, and esophagus [5]. Common symptoms of CD include abdominal pain, diarrhea, fatigue, and weight loss [6]. Long-term complications as a result of uncontrolled inflammation can include intestinal obstruction and enteric or perianal fistulas, which often require surgery [5, 7].

Rectal bleeding, stool frequency, and abdominal pain are common measures used to define disease activity in UC and CD [8]. Bowel urgency, the sudden and urgent need to have a bowel movement, is also an important factor to consider, but it is often overlooked as a measure of ongoing disease activity [8, 9]. Yet, bowel urgency has been reported as the most important/distressing symptom to patients with UC [10–12] and affects 60–80% of them [13, 14]. Further, bowel urgency is associated with reduced quality of life and future risk of hospitalizations, corticosteroid use, and colectomy in UC [15]. Bowel urgency is also a commonly reported symptom in CD: two-thirds of patients with CD experience bowel urgency, and in half of these, it is moderate to severe [14]. In both UC and CD, moderate-to-severe bowel urgency is associated with increased bowel movements and stool frequency, as well as abdominal pain [14]. Although bowel urgency is not included in most current indices of disease activity for inflammatory bowel disease [9], its control is strongly associated with improved quality of life and clinical measures of disease activity [16]. Therefore, bowel urgency may be a relevant disease marker and treatment target for UC and CD [8].

Bowel urgency can be assessed using the Urgency Numeric Rating Scale (UNRS), a single-item measure originally developed for measuring patient-reported bowel urgency in UC [17]. The UNRS asks respondents to rate the severity of their bowel urgency over the last 24 h on a scale of 0 for no urgency to 10 for worst possible urgency [17]. In an analysis of phase 3 data from patients with moderate-to-severe UC, the UNRS demonstrated strong test-retest reliability, known-groups validity, convergent and discriminant validity, and responsiveness to change [18]. In a subsequent mixed-methods study involving qualitative and quantitative investigations, the UNRS was also found to be a valid and reliable tool for assessing bowel urgency in CD [19].

Although bowel urgency is a well-established issue in UC, less is known about its importance to patients with CD. Further, what level of bowel urgency control patients with UC or CD would consider to be sufficient or in remission is not clear. Here, we describe the results of a qualitative interview study investigating how adult patients with moderately to severely active UC or CD think about the concept of bowel urgency and, using the UNRS, the highest level of bowel urgency they would consider to be remission.

## Methods

### Study design

This was a cross-sectional, qualitative, observational study in which participants engaged in a single semi-structured, telephone interview exploring how they describe different severity levels of bowel urgency, including remission. Discussions were anchored to the UNRS to understand how patients with moderately to severely active UC or CD use this scale to rate different bowel urgency severity levels. This patient population was targeted as it reflects the study population of the clinical trial program in which the UNRS was being utilized. This population was also targeted as previous literature suggests that bowel urgency is a common symptom among patients with moderately to severely active UC or CD [14]. Interviews were conducted between April and July 2023.

### Recruitment of participants

Eight clinical sites in the US were asked to recruit approximately 40 adults (≥ 18 years of age), including approximately 20 with UC and 20 with CD, across diverse sociodemographic characteristics (e.g., gender, race, and educational level). Participants were required to have a physician’s diagnosis of moderately to severely active UC or CD at least 6 months prior to screening (based on laboratory examination of blood and/or stool matter, X-ray, or endoscopic examinations), currently be taking biologic and/or conventional therapy for UC or CD, and to self-report experiencing or having experienced UC- or CD-related bowel urgency in the past 3 months. Participants could not have ever had an ileostomy, colostomy, intra-abdominal surgery, or other surgery for the treatment of UC or CD; any other conditions affecting the digestive tract that may confound the discussion of UC or CD symptoms or impacts; or any other relevant current medical condition that may interfere with giving consent or completing the study procedures. Participants had to be able to understand, read, and speak English sufficiently to complete the assessments and the study interview, be willing and able to attend a telephone interview session, and be willing have the interview audio recorded.

Site staff reviewed medical records to identify potentially eligible patients and used a standardized script for screening. All sites and site principal investigators underwent qualification checks. Eligible patients who agreed to participate were sent a cover letter, the UNRS, and a sociodemographic questionnaire; these documents were provided in a sealed envelope with instructions not to open it before the interview. All recruitment procedures complied with regulations in the US Health Insurance Portability and Accountability Act.

### Interview procedure

Phone interviews of up to 90 min were conducted by six experienced qualitative interviewers, each of whom conducted 1 to 13 interviews. Interviewers were specifically trained for this study by reviewing the interview guide as a group, focusing on the objectives for each question and anticipated responses, and participating in a mock interview. At the beginning of each interview, the interviewer fully explained the study to the participant and reviewed the informed consent form. All participants provided consent prior to their interview, via DocuSign or by signing and returning a paper consent form in the mail, if preferred. The interview proceeded using a semi-structured interview guide. The first (concept elicitation) portion of the interview explored symptom and impact experience, focusing on bowel urgency and bowel urgency remission. The second (cognitive) portion instructed participants to complete the UNRS and then asked them to discuss severity of bowel urgency using the 0–10 response scale. Participants also completed the sociodemographic questionnaire, and clinical sites completed clinical case report forms to provide descriptive data (e.g., date of diagnosis) for the sample. All data were recorded and tracked by unique participant identification numbers.

### Ethics

All participants provided electronic or written informed consent. The study protocol and recruiting clinical sites were approved by Advarra (Columbia, MD), a central institutional review board (study reference: Pro00069827).

### UNRS

The UNRS is a single-item measure that asks respondents to rate the severity of their bowel urgency in the previous 24 h, using a scale of 0 for “No urgency” to 10 for “Worst possible urgency.”

### Analysis

Audio files were transcribed and transcripts were analyzed using ATLAS.ti version 22.0 or higher (ATLAS.ti Scientific Software Development GmbH, Germany). Qualitative research outputs were analyzed using a content thematic analysis approach [20]. Researchers first reviewed the interview transcripts to familiarize themselves with the dataset. Quotes were then selected and assigned to codes to identify emerging themes, and the data were queried based on the research objectives. Codes were merged into higher data themes, split into more nuanced themes, and/or reassigned/reorganized to answer the research questions. A coding dictionary was developed by a lead investigator based on the interview guide and the themes and concepts that emerged during the first few interviews. Coding of transcripts was conducted by four investigators who were trained to use the coding dictionary. After all coding was completed, a final quality check of each transcript was conducted. The data were tabulated to quantify the findings and the frequency of the different themes. For quantitative analyses, only descriptive statistics were calculated.

## Results

### Participant characteristics

The study included 40 participants (*n* = 19 UC, *n* = 21 CD) recruited across six US clinical sites (*n* = 2–14 per site) (Table 1). Mean age was 48 years (standard deviation [SD], 17; range 20–84) for UC and 45 years (SD, 15.4; range 20–69) for CD. Most participants were female (63% for UC, 71% for CD), not Hispanic or Latino (84% for UC, 95% for CD), and White (74% for UC, 91% for CD). More than half of participants with UC were married (63%) and employed full time (58%), whereas less than half of the participants with CD were married (38%) and employed full time (48%). The mean time since diagnosis was 11 years (SD, 8) for participants with UC and 10 years (SD, 11) for those with CD. Participants with UC reported experiencing bowel urgency for a mean of 10 years (SD, 9), and those with CD reported experiencing it for a mean of 15 years (SD, 14); therefore, most participants had likely experienced bowel urgency at least since their diagnosis. Mean UNRS scores were 4.3 (SD, 3.1) for participants with UC and 5.2 (SD, 3.3) for those with CD.

**Table 1:** Sociodemographic and clinical characteristics of participants

Characteristic	UC *N* = 19	CD *N* = 21
Age (years), mean (SD) [range]	48 (17) [20–84]	45 (15) [20–69]
Gender, *n* (%)		
Male	7 (37)	5 (24)
Female	12 (63)	15 (71)
Non-binary or other^a^	0 (0)	1 (5)
Ethnicity, *n* (%)		
Hispanic or Latino	3 (16)	1 (5)
Not Hispanic or Latino	16 (84)	20 (95)
Racial background, *n* (%)^b^		
Black or African American	3 (16)	1 (5)
White	14 (74)	19 (91)
Other^c^	1 (5)	0 (0)
Multiple^d^	1 (5)	1 (5)
Marital status, *n* (%)^b^		
Single	5 (26)	7 (33)
Married	12 (63)	8 (38)
Divorced	1 (5)	6 (29)
Widowed	1 (5)	0 (0)
Employment status, *n* (%)^b^		
Employed, full-time	11 (58)	10 (48)
Employed, part-time	1 (5)	2 (10)
Homemaker/housewife	0 (0)	1 (5)
Unemployed	0 (0)	1 (5)
Stay-at-home parent	1 (5)	0 (0)
Retired	4 (21)	3 (14)
Disabled	2 (11)	4 (19)
Highest education level, *n* (%)^b^		
Secondary/high school	5 (26)	6 (29)
Some college	9 (47)	7 (33)
College degree	3 (16)	6 (29)
Post graduate degree	2 (11)	0 (0)
Other^e^	0 (0)	2 (10)
Time since diagnosis (years), mean (SD)	11 (8)	10 (11)
Participant-reported duration of bowel urgency (years), mean (SD)^f^	10 (9)^g^	15 (14)^g^
UNRS score, mean (SD)^h^	4.3 (3.1)	5.2 (3.3)

*CD* Crohn’s disease, *SD* standard deviation, *UC* ulcerative colitis, *UNRS* Urgency Numeric Rating Scale

^a^Prefers to self-describe: “trans female” (*n* = 1, CD)

^b^Numbers do not add to 100%, due to rounding

^c^Other race: Hispanic (*n* = 1, UC)

^d^Multiple races: “White and Hispanic” (*n* = 1, UC), “Indian and White” (*n* = 1, CD)

^e^Other includes Trade School (*n* = 1, CD), Votech (Cosmetology) (*n* = 1, CD)

^f^Duration of bowel urgency was reported by participants during the interview. Mean and SD were calculated using the average of ranges when participants provided a range rather than a discreet number (e.g., “4.5” was used in calculations for a participant reporting they had experienced bowel urgency for “4 to 5 years”)

^g^Data from 16 participants with UC and 19 participants with CD; three participants with UC and two with CD were considered missing due to providing unclear responses

^h^All participants were asked to complete the UNRS at the start of the cognitive debriefing portion of the interview

### Terminology used to describe bowel urgency

Among the 19 participants with UC, nearly three-quarters (*n* = 14) used terms relating to the frequency and immediacy of bowel urgency (Table 2). Most described the need to find a bathroom immediately (*n* = 9) or used the words “urgent” or “very urgent” (*n* = 3). Almost one-third of participants with UC (*n* = 6) described an association between urgency and other bowel symptoms; of these, the most common was bleeding (*n* = 2). Over half of participants with UC (*n* = 12) described their bowel urgency as being associated (co-occurring) with abdominal pain. These included “stomach cramping” and “abdominal pain/stabbing abdominal pain/painful” (*n* = 6 each).

**Table 2:** Terminology used for the concept of “bowel urgency”

General categories of descriptions	*N* (%)	Illustrative quotes
Associated with pain in abdomen	UC: 12 (63%)	“With me, it starts like with my stomach cramping, and it just feels like I have to pass gas, but I don’t know which one. It could go either way.”
	CD: 14 (74%)	“In the very beginning it was like my stomach felt like it was literally on fire. And it was a constant on fire.” “I would describe it as kind of a pressure in my abdomen and kind of not exactly hot, but it’s a little warmer around the bottom area.”
Associated with immediacy and/or frequency	UC: 14 (74%)	“It feels like I start to get a stomach ache and then I have to run to a bathroom…” “It’s awful. It gets so gassy and you’ve got to go now… you can’t even hold it back.”
	CD: 12 (57%)	“It feels pretty bad. It feels like…you need to get to a bathroom quickly. It’s…kind of overwhelming…if you’re really far away, I mean, there’s always the chance…you may not be able to hold it… you need to get to the bathroom. So, that urgency to go without going in your pants is pretty, pretty strong.” “For lack of a better word, it’s like you just need to get out of my way. It’s not pleasant. Sometimes if you have to go to the bathroom, you can hold it. That’s not even an option. Like you just can’t”
Associated with related bowel symptom	UC: 6 (32%)	“It’s like that feeling like you really have to pee and you’re on a road trip, but you don’t know where the bathroom is, on steroids, because if you pee yourself, it’s not as embarrassing as if you poop your pants. It’s like an all-consuming thought and physical response of your body to be like, get me to a bathroom, get me to a bathroom.”
	CD: 7 (33%)	“Just have to hurry up and barely make it… you’d have to hurry up and go. It would just be watery, like all mostly watery. And bloody, blood, a lot of blood. Well, it was blood when I wiped. And then it got worse in the last month or two. It would just be blood in the commode.”
Other	UC: 3 (16%)	“… It’s like a heavy sensation in my lower gut. And I have learned that not to ignore that heavy sensation. I made the mistake of ignoring it a couple times. So basically, I feel that heavy sensation, and then instantly, I am unable to contain what is there.”
	CD: 3 (14%)	“It’s sprint to the bathroom or you’re going to have to change your clothes, and it’s super embarrassing.” “Desperate. Extremely awful. I would sometimes not go places for a fear that I would have an accident, or I’d have to rush to a bathroom, or a bathroom wouldn’t be available.”

Participants were asked “How would you describe this urgency or sudden need to have a bowel movement? What does it feel like?” *CD* Crohn’s disease, *UC* ulcerative colitis

Two-thirds (*n* = 14) of the 21 participants with CD used terms relating to abdominal pain to describe their bowel urgency experience (Table 2). Some of the most common terminology used by these participants was related to pain (*n* = 6), stomach cramping (*n* = 5), and pressure in the abdomen (*n* = 3). Notably, over half (*n* = 12) used terms relating to the immediacy they felt when bowel urgency was present. Among participants with CD, the most frequent description used was needing to find a bathroom immediately (*n* = 8). One-third of participants with CD (*n* = 7) described other associated bowel symptoms. Of these, the most common description was a warm/tingling sensation around the bottom area (*n* = 3).

### Impact of bowel urgency

Nearly two-thirds of the 19 participants with UC (*n* = 12) reported impacts on work/productivity due to bowel urgency, including having to quit their job, retire early, and/or apply for disability or family/medical leave (*n* = 6); needing workplace concessions (*n* = 3); having difficulty completing work tasks (*n* = 2); and having to pack extra clothes in case they had an incident of fecal incontinence (*n* = 2) (Table 3). One participant with UC reported that productivity at home was impacted by bowel urgency. Social impacts of bowel urgency were endorsed by just over half of the participants with UC (*n* = 10), including difficulty with socializing with friends (*n* = 6), an inability to travel (*n* = 3), a general lack of social interactions (*n* = 3), difficulty in going out in public places (*n* = 2), and difficulties camping with friends and family (*n* = 2). One participant reported being unable to leave the house during a flare, and another reported having difficulty taking care of children due to bowel urgency. About one-third of participants (*n* = 6) reported mental or emotional impacts of bowel urgency, including feeling anxious (*n* = 4) or needing to plan excessively (*n* = 3). About one-quarter reported physical impacts of urgency (*n* = 5), the most frequent of which was being unable to exercise (*n* = 3). One participant each reported a lack of energy, inflammation, and sleep disruption. Five participants with UC identified other types of impact, including needing to stay close to a bathroom frequently (*n* = 3), having difficulty grocery shopping due to being away from home (*n* = 2).

**Table 3:** Impact of bowel urgency

Impact	*N* (%)	Illustrative quotes
Social/relationship	UC: 10 (53%)	“I went to a concert early this summer and I spent the whole time in the porta potties and standing by the porta potties the whole concert and missed most of the concert… And then I haven’t been able to go on camping trips or anything with friends because I don’t have access to the bathroom… for instance, since it’s summer time and I have two kids, it’s been fun to take them to like the pool and the lake, but it’s been difficult. I have to have people come with me…I have to make sure that we’re close to a bathroom, so I usually can’t go to the lake anymore. But if we go to the pool, I have to make sure I’m close to the bathroom, and that they can watch my kids…”
	CD: 16 (76%)	“I don’t want to go out places because you never know when that urgency’s going to hit, If you eat certain foods, you don’t know how you’re going to react to it. So it kind of took a toll, made you want to be more of a recluse, stay home, stay close to where you’re comfortable going to the bathroom. It’s just kind of unpredictable.”
Mental/emotional	UC: 6 (32%)	“Yeah. Everyone says don’t stress out, but when you have ulcerative colitis and everything you’re going through, it really, it’s really stressful, and it definitely impacts your mental health as well as your physical health.”
	CD: 12 (57%)	“It definitely is anxiety inducing if I can’t, if I don’t know there’s like a public bathroom if I’m in public. Or if I’m somewhere I need to be at work or something, it’s stressful knowing that I might need to use the restroom like super quickly, and that might not be an option for me.”
Work/productivity	UC: 12 (63%)	“I had to quit work when I was 24 because I was going in and out of work towards the end. It affected me, like if it wasn’t under control, it could last anywhere from a day up to 10 days, sometimes longer, just depending. And so I ended up having to quit work because I developed like really bad depression and anxiety from it. Not just from that, but other things as well, but mostly it was that.”
	CD: 12 (57%)	“… I’d get a new job and I would have to rush to the bathroom and they couldn’t allow me. They were like, we have to find somebody to cover you, and I’m like, you don’t understand. I’ve got to go right now. One time I had an accident at a place and they considered it hazardous, so they let me go. There was one that was like, no, you have to wait, you have to wait, you have to wait. And it was like, no, I have to go. So I was let go because I left my station because they needed somebody to cover me.”
Physical	UC: 5 (26%)	“Going to the gym, that was terrible. Grocery shopping or a long day of being out of the house was not ideal. And then any like parties or anything where like I wanted to drink but I knew alcohol would be a trigger for me, so that was also challenging too. Road trips, not a fun time. Flying was also not an option then.”
	CD: 9 (43%)	“When urgency is a problem, definitely activities that require of me to be active. So any exercise. I wouldn’t really say walking around, but anything that causes me to have to move the body a lot would definitely cause – if I had a flare up I wouldn’t really be able to do that.”
Other	UC: 5 (26%)	“… I can’t go out like normal people to go out and eat in a restaurant, like everybody… I can’t go out and do none of that because not only do I have to be in the bathroom, but I can’t eat half of the stuff that’s there. That’s not fair to me. It’s not fair that I have to live this different life. Everything got to be gluten free. You don’t know this, no seeds. And when you break it down, no red meat. So what am I supposed to eat? Can’t eat oranges. It’s a nightmare for me every day… this is a nightmare I’ve lived with since a child.”
	CD: 8 (38%)	“… You have to pack a bag of underwear and clothes and wipes and stuff, to take any time you go anywhere. Even if you’re going down the road, you have to make sure you’re taking all this with you everywhere you go.”

Participants were asked “Briefly, how has that urgency impacted your daily life? Are there any activities that are more difficult for you to complete when urgency is a problem for you, for example?” *CD* Crohn’s disease, *UC* ulcerative colitis

Of the 21 participants with CD, about two thirds (*n* = 16) endorsed social impacts of bowel urgency (Table 3). Many participants (*n* = 8) reported difficulty traveling (e.g., inability to drive long distance or a need for more stops). Difficulty socializing with friends and having a hard time going to public places/events (e.g., family sporting games and restaurants) were each reported by five participants. Four participants endorsed the notion that bowel urgency impacts all aspects of socialization, and two had difficulty engaging in leisure activities in general. Mental or emotional impacts of bowel urgency were reported by 12 participants with CD. These included a need for excessive planning (*n* = 6), feeling anxious (*n* = 4) or depressed (*n* = 3), and embarrassed (*n* = 2). Impacts on work/productivity due to bowel urgency were also reported by 12 participants. The most frequent were difficulty completing work tasks (*n* = 4); missing work (*n* = 3); needing to quit their job, retire early, and/or apply for disability due to their bowel urgency (*n* = 2); or needing workplace concessions (*n* = 2). Physical impacts because of bowel urgency were reported by a little under half of participants with CD (*n* = 9), including dietary restrictions (*n* = 5), being unable to exercise (*n* = 4), and having difficulty sleeping (*n* = 2). Other types of impact were reported by eight participants with CD, including needing to stay close to a bathroom frequently (*n* = 4).

### Stool frequency and bowel urgency severity

**Fig. 1:**
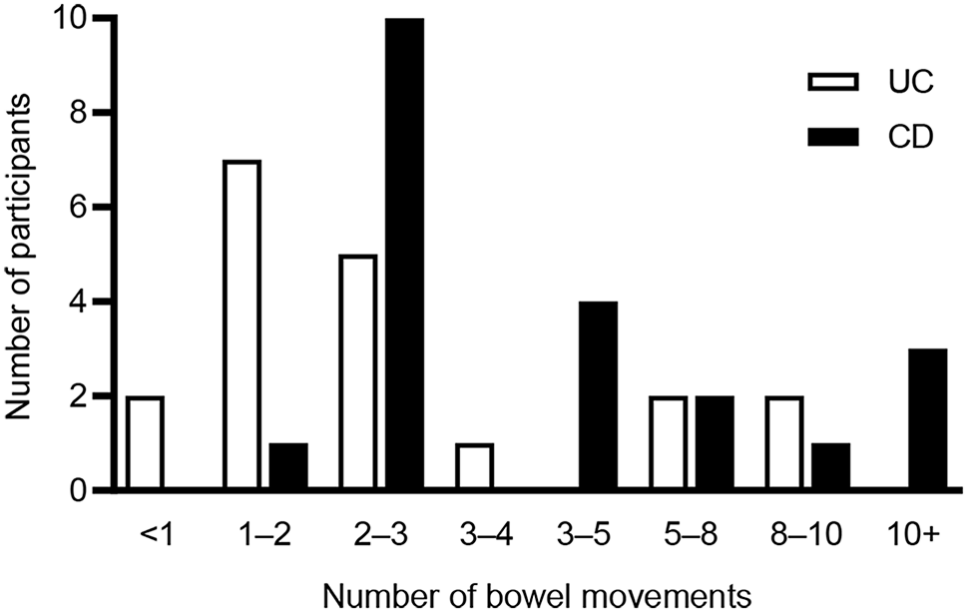
Number of bowel movements experienced in a “typical” (non-flare) day. Participants were asked “About how many bowel movements do you have in a usual day?” If it varied, they were asked to describe the range and then focus on the past 24 h. Most participants spontaneously provided a range for the number of bowel movements they experience per day. Therefore, the presented ranges overlap. *CD*, Crohn’s disease, *UC* ulcerative colitis

Participants in both groups were asked about the frequency of bowel movements in a “typical” (non-flare) day (Fig. 1). Among participants with UC, one or two bowel movements per day were reported most frequently (*n* = 7). Among participants with CD, two or three bowel movements per day were reported most frequently (*n* = 10).

Some participants described the number of bowel movements they would experience during a flare or a more severe day. Five participants with UC reported experiencing from five to six bowel movements per day (*n* = 1) to as many as five per hour (*n* = 1); the remaining three participants reported 10–12 bowel movements per day, up to 20 per day, and “going throughout the day” (*n* = 1 each). Nine participants with CD reported from five to six (*n* = 2) to as many as 20 bowel movements per day (*n* = 1); the remaining six participants reported up to eight (*n* = 2), up to 10 (*n* = 2), up to 12 (*n* = 1), or up to 16 bowel movements per day (*n* = 1).

Study participants were asked to describe how frequently they experienced bowel urgency (Table 4). Among participants with UC, six reported experiencing bowel urgency with every or almost every bowel movement. Four described the frequency of bowel urgency as a percentage of bowel movements that have urgency (50% for *n* = 1, 75% for *n* = 2, and 90% for *n* = 1). Some participants experienced bowel urgency one to three times per week or explained that their frequency of bowel urgency varied depending on whether they were experiencing a flare (*n* = 3 each). Among participants with CD, some reported that their frequency of bowel urgency varied depending on whether they were experiencing a flare and that their bowel urgency was infrequent when they were in remission (*n* = 5). Another four participants with CD described their frequency of bowel urgency as a percentage of bowel movements that involve urgency (50% for *n* = 3, 80% for *n* = 1). Some participants with CD reported experiencing bowel urgency 1 to 3 days per week, one to three times per day, or explained they had bowel urgency with every or almost every bowel movement (*n* = 4 each).

**Table 4:** Frequency of bowel urgency

Frequency	*N* (%)	Illustrative quotes
With every or nearly every bowel movement	UC: 6 (32%)	“Oh, most of the time. Yeah, like every time. Yeah, every time.”
	CD: 4 (19%)	INTERVIEWER: “… How many bowel movements do you have in a usual day?” PARTICIPANT: “Twelve to 13.” INTERVIEWER: “… How often do those like Crohn’s disease related urgencies to have a bowel movement occur?” PARTICIPANT: “About the same.” “Literally almost every day. I can’t tell you a day probably since March where I haven’t had it at least five times.”
Varies depending on whether in flare or not	UC: 3 (16%)	“When I have a flare up. It’s pretty bad. Like, when I’m in the middle of a flareup, it’s literally probably like six to eight times a day.”
	CD: 5 (24%)	“During the flareups, it’s like 85% of my day. But when I feel like I’ve got it under control, it’s, I don’t know, 15 to 30 [percent].”
One to three days per week	UC: 3 (16%)	“So today, this year versus - today with this medication, maybe the somewhat of an urgency is maybe once a week.”
	CD: 4 (21%)	“I would say maybe, I don’t know, you might get once out of a week or twice out of a week where you have a bad day. Maybe three times a week.”
One to three times per day	UC: 0 (0%)	–
	CD: 4 (19%)	“At least two times, two to three times a day. It’s like it varies, based on how much I consume for a food. If I just do a snack, I’ll be fine. But if I eat a meal, it start to affect it. It seemed like I digested my food little faster and then I need the bathroom real quick, which is annoying.” “Probably maybe two of them, but always the first one. In the morning, after I ate breakfast, that was always, I’d go to Walmart or something and I’d be searching for the bathroom, running, because the store’s so big and I’m like, oh God, I’m so far from the bathroom. And I’d take off, trying to get there in time and it was a gamble.”
With 50% of bowel movements	UC: 1 (5%)	“I think 50% would be a good number.”
	CD: 3 (14%)	“In a week’s time period, I would say half of the week would be, yeah, definitely having to run somewhere.”
With 75–80% of bowel movements	UC: 2 (10%)	“I would say 75% in the morning, I better get in there. Once the pain starts, and I have to be there. Yeah.”
	CD: 1 (5%)	“I’d say about 80% of the time.”
Less than once a week	UC: 2 (11%)	“With the flares is, I guess that’s a little bit different because I’m having to cramping and I’m having more of the like constipation where I’m bleeding when I’m trying to go to the bathroom. I say the urgency maybe once a month. And that’s like maybe after I ate something that my stomach didn’t totally agree with.”
	CD: 0 (0%)	–
With 90% of bowel movements when medicine is not working	UC: 1 (5%)	“With the medicine, if a biologics not working, I would say nine – 90% of them are urgent, got to go.”
	CD: 0 (0%)	–
Only when experiencing severe stress	UC: 1 (5%)	“Now it is isolated to moments of stress. And I mean like pretty severe stress, not just your average…. Oh my God, I got to go to the bathroom. It was, it’s very isolated to like severe moments of stress.”
	CD: 0 (0%)	–

Participants were asked “How often do you have UC/CD-related urgency to have a bowel movement?” If it varied, they were asked to describe the range and then focus on the past 24 h. *CD* Crohn’s disease, *UC* ulcerative colitis

Study participants were also asked what terminology they used to describe the severity of their bowel urgency (Table 5). For participants with UC, terms related to the amount of time they have to get to the bathroom before having an incident of fecal incontinence or having to hurry to the bathroom were also the most often used (*n* = 11). Others (*n* = 7) described the symptoms or sensations they experience when thinking about severity of bowel urgency, including references to blood or bleeding (*n* = 2) and stomach pain or cramping (*n* = 2). A few described the severity of bowel urgency in terms of the frequency of urgent episodes (*n* = 3) or used terms like “bad” or “severe” (*n* = 3). Participants with CD most often used terms related to the amount of time they have to get to the bathroom before having an incident of fecal incontinence (*n* = 9). Others referenced symptoms or sensations (such as pain or cramping) when experiencing bowel urgency (*n* = 5) and described the severity of their bowel urgency in terms of mental and/or physical impacts (*n* = 5). Some participants with CD (*n* = 4) used the term “flare” to describe the severity of their bowel urgency.

**Table 5:** Terminology used to describe the severity of bowel urgency

Severity	*N* (%)	Illustrative quotes
Need to find bathroom now/ hurry/not going to make it	UC: 11 (58%)	“It’s, oh gosh it’s terrible. It comes on so quickly and you only have moments to make it to the bathroom. And sometimes I don’t even have time to open the toilet lid before it starts just coming out. And then sometimes it’s just straight blood and it’s just, it’s hard. You can’t control, you can’t hold it in.” “Here’s what I say. I got to go. I got to go to the bathroom right now. I got to go, I got to go potty… When I say now, it’s now, because I’m going to potty on myself.”
	CD: 9 (43%)	“It’s more of a when you’ve got to clench up your bottom and then then move with haste, like very fast urgency…” “… I don’t know if I’m going to make it. Just horrible… I just had to go to the bathroom so incredibly bad. It was like pushing people out of the way type of thing… When I’m in the moment, it’s all about get there, get to the bathroom. I’m not saying anything. I’m just like, oh my God…”
Described in terms of symptoms/sensations	UC: 7 (37%)	“… It’s a horrifying feeling… sensation, horrifying sensation.”
	CD: 5 (24%)	“I would say that I sometimes I do feel rushed to go to the restroom. There is times where I’ll get a kind of heat like pain in my abdomen. So it feels very hot, I guess.” “… I can usually tell by the level of pain that I’m in… Less pain is I could probably walk slow to the bathroom instead of like sprinting.” “… the flareups, it’s, I don’t want to use the word deadly because it’s not deadly, but it is severe. It’s severe pain, it’s severe bloating, obnoxious gas. And it’s just way over the top.”
Bad/bad day/severe	UC: 3 (16%)	“I usually just say like it’s bad or painful… some days are like really bad, like some days I just want to stay home and just be in bed and close to a bathroom.” “Just not feeling it, having a bad, bad day.”
	CD: 2 (10%)	“I’d just say bad. And I think that too is because of the length of time I’ve had it and me living with it, you get used to, okay, this is just the way it is. Because when people say do you have normal bowel movements? I’m going, I don’t even know what normal is anymore.”
Flare	UC: 1 (5%)	“But I guess to me a flares is a flare, severity wise.”
	CD: 4 (19%)	“No, we usually just, I usually classify it as a flareup, just because it just acts up sometimes, and then can I be good one day and then perfectly like and be in bed, stuck in bed the next, even whenever I’m experiencing a flareup.” “No, just having a flare up. I know if I’m down for the count if I’m having a flare up. If it’s acting up, I just, you know.”
Described in terms of impact	UC: 0 (0%)	–
	CD: 5 (24%)	“It’s defeating, I guess. I’m getting to the point where I’m just tired of being, having these issues. I don’t know how to describe that, how I would describe the urge. It’s just, you just have to be like – I don’t know how to describe that one. I’m sorry.” “I actually don’t eat all day long. And I think that’s why after the morning is over, and I went to the bathroom so many times. When I get to work, I don’t eat. Because I know as soon as I eat something, it’s going to go right through me, and I’m going to be back into the bathroom.”
Described in terms of frequency	UC: 3 (16%)	“But the amount of times is what, I guess is how I distinguish it.”
	CD: 0 (0%)	–

Participants were asked “What words or terms do you use to describe how bad your urgency is? In other words, how do you talk about different levels of severity of your urgency, from not having any to the point of having an accident?” *CD* Crohn’s disease, *UC* ulcerative colitis

When asked if they ever had an incident of fecal incontinence in the context of their bowel urgency, only four of the participants with UC reported never having one. The 15 who had ever (since their diagnosis) experienced incidents reported from one to two incidents (*n* = 6) to approximately 10 (*n* = 2); others reported three to six incidents ever (*n* = 4). Among participants with CD, only three reported never having an incident. Of the 18 who had experienced incidents, a third (*n* = 6) reported that their fecal incontinence episodes were ongoing or frequent. Some participants with CD reported six incidents in the past month (*n* = 2), “a few” incidents per year (*n* = 3), one to five incidents ever (*n* = 6), or 20–30 incidents ever (*n* = 3).

### Bowel urgency remission

Study participants were specifically asked if they had ever heard of the term “remission” in relation to their bowel urgency symptom and/or in the context of their overall UC/CD condition (Table 6). Among participants with UC, most reported having heard the term “remission” regarding their overall condition (*n* = 14) and/or regarding their bowel urgency symptom specifically (*n* = 13). Most participants (*n* = 18) defined remission of bowel urgency as “feeling normal” with fewer bowel movements and reduced levels of bowel urgency. Other common definitions of bowel urgency remission included having a “good day” (*n* = 4), a period where they are experiencing no or minimal pain (*n* = 4), a state when they are more relaxed and able to “hold” their bowel movements in (*n* = 3), or a time they can eat normally (*n* = 2). Two participants with UC spontaneously used the term “remission” to describe bowel urgency getting better. Most participants with CD had heard the term “remission” with respect to their overall condition (*n* = 17) and/or their bowel urgency symptom specifically (*n* = 14) (Table 6). Most (*n* = 18) defined remission of bowel urgency as a state with a normal or reduced number of bowel movements and bowel urgency either absent or less frequent. Other common definitions of bowel urgency remission included: having a “good day” (*n* = 6), having minimal or no pain (*n* = 6), not feeling rushed to use the toilet (*n* = 5), having a period where they can eat normally (*n* = 4), or having times when they are able to do more activities (*n* = 2). Three participants with CD spontaneously used the term “remission” to describe bowel urgency getting better.

**Table 6:** Terminology used to describe bowel urgency remission or times with normal or reduced bowel movements

Terminology	*N* (%)	Illustrative quotes
Normal or reduced number of bowel movements; Bowel urgency gone or less frequent	UC: 18 (95%)	“… Because I’m in this study and I have to answer all this to an app, I can see if I have a better day, I don’t have as many bowel movements or as many urgencies.” INTERVIEWER: “And would you use a specific word to say my urgency is controlled?” PARTICIPANT: “It’s just not as many. It’s still not controlled. It’s just not as many, as frequent.”
		“… I can go out to eat, and then after I can go shopping for like two hours with my friends. Like I feel like a normal human being.”
		“Going to the bathroom normally, regularly, no diarrhea, no being constipated. I didn’t have any issues, like I wasn’t bleeding.”
		“… When I start being more of a normal person again, that’s sort of my definition of me being in remission, that I’m now living more of a normal life than I did when the UC was at its worse.”
	CD: 18 (86%)	“I’m satisfied. I feel normal. Normal, yeah. You can eat and actually enjoy afterwards, you don’t have to be worried about scouting out, finding places to go.”
		“Now that I’ve been out, I would say I probably only went three times a day… I ate a little bit more today, but okay, when it’s good, like now, it’s like maybe three, four times, which is probably normal…”
		“I felt, literally for over a year, I’ve had normal bowel movements with no urgency, nothing abnormal with my stool, things like that. It was like I was a normal person again. Literally had no symptoms at all. It was great…”
“Good day”; better	UC: 4 (21%)	“… Meaning that this day I’ve only went to the bathroom, let’s say, wow, it’s eight o’clock at night and I only went to the bathroom six times today, or seven. This is a good day…”
		“… Having more good days than bad days. And I would call that remission. Or having weeks, which I’ve had a week or two here and there that are, I would call in remission…”
	CD: 6 (29%)	“… Just having a good day, doing good. I’ll even get pretty excited, like I had a solid poop. It was an actual turd. Oh, it’s like time to celebrate, like break out the champagne.”
No or less pain	UC: 4 (21%)	“… When you have the pain, it gives you a pain and then the pains go away a little bit. And then some of it come back again… But when pain, it happens inside, maybe a little pest a little while, and then it’s, it is, it’s not painful…”
	CD: 6 (29%)	“Just I feel normal. Like I don’t have abdominal pain, I don’t have flareups, or like very minimal flareups. I don’t have like diarrhea or anything like that. It’s just like normal bowel movements.”
More relaxed; not rushed; able to wait to use toilet (able to “hold it”)	UC: 3 (16%)	“Probably just, I don’t know, almost, it’s just a weird word for it, but almost like relaxation because I’m not so stressed out, if that makes sense.”
		“… Kind of like you can hold it and you can handle it a little bit.”
	CD: 5 (24%)	“I know that I need to use the restroom, but I feel a lot more relaxed and that, I can just walk down to the restroom. I don’t feel like I have to run or rush to get everything done. And my body doesn’t feel hot or anything. It just feels very normal.”
Emotion/sensation (wonderful; relieved; lucky; energetic; freeing; calm; thankful; not bothered)	UC: 4 (21%)	“I just feel like relief and better. I usually—so my anxiety isn’t as high to find a bathroom and stuff, so it’s just a little bit of a relief for my anxiety too.”
		“I would say calm. Calm feeling, remission.”
		“Thankful really, to be honest with you. Thankful that I’m not flared that particular day or time.”
	CD: 5 (24%)	“Pretty good. For the most part, I feel, you know, energetic…”
Can eat normally	UC: 2 (11%)	“… Maybe I can actually go for a two-hour ride to go to a beach without having to stop in the woods. Being able to have myself a good fruit salad, without worrying about anything else, the pain and the things that I go through when I’m not in remission, to feel peaceful…”
	CD: 4 (19%)	“I would need to be able to eat and not get sick within five minutes of eating or while I’m eating.”
Bowel urgency remission	UC: 2 (11%)	“That’s what I would say it is. I have got over a plateau of being on remission with the medication that makes me not feeling that urgency anymore. So yeah, remission is a good word.”
	CD: 3 (14%)	“It’s in remission or I’m not having a flareup. You know, I’m normal for the day. I’m good.”
Can do more activities	UC: 1 (5%)	–
		“That I feel great and that I’m usually more able to do more activities.”
	CD: 2 (10%)	“You have less bowel movements. You’re able to go out and do things, I guess, without worrying. But you still worry because you don’t know if they’re going to have that flare up…”
“Controlled”; settled down	UC: 3 (16%)	–
	CD: 1 (5%)	“Controlled is a great word. Settled down, regulated, steady.”
“Something went to sleep”	UC: 0 (0%)	–
	CD: 1 (5%)	“To me, remission means something went to sleep. It’s still there, but it’s in a deep sleep. So it could actually come back if you get yourself into a situation where you’re under an intense amount of stress or hardship of some sort…”

Participants were asked “What term do you use to describe times when your urgency gets better or is “controlled?”” *CD* Crohn’s disease, *UC* ulcerative colitis

### Mapping of bowel urgency on the UNRS

**Fig. 2:**
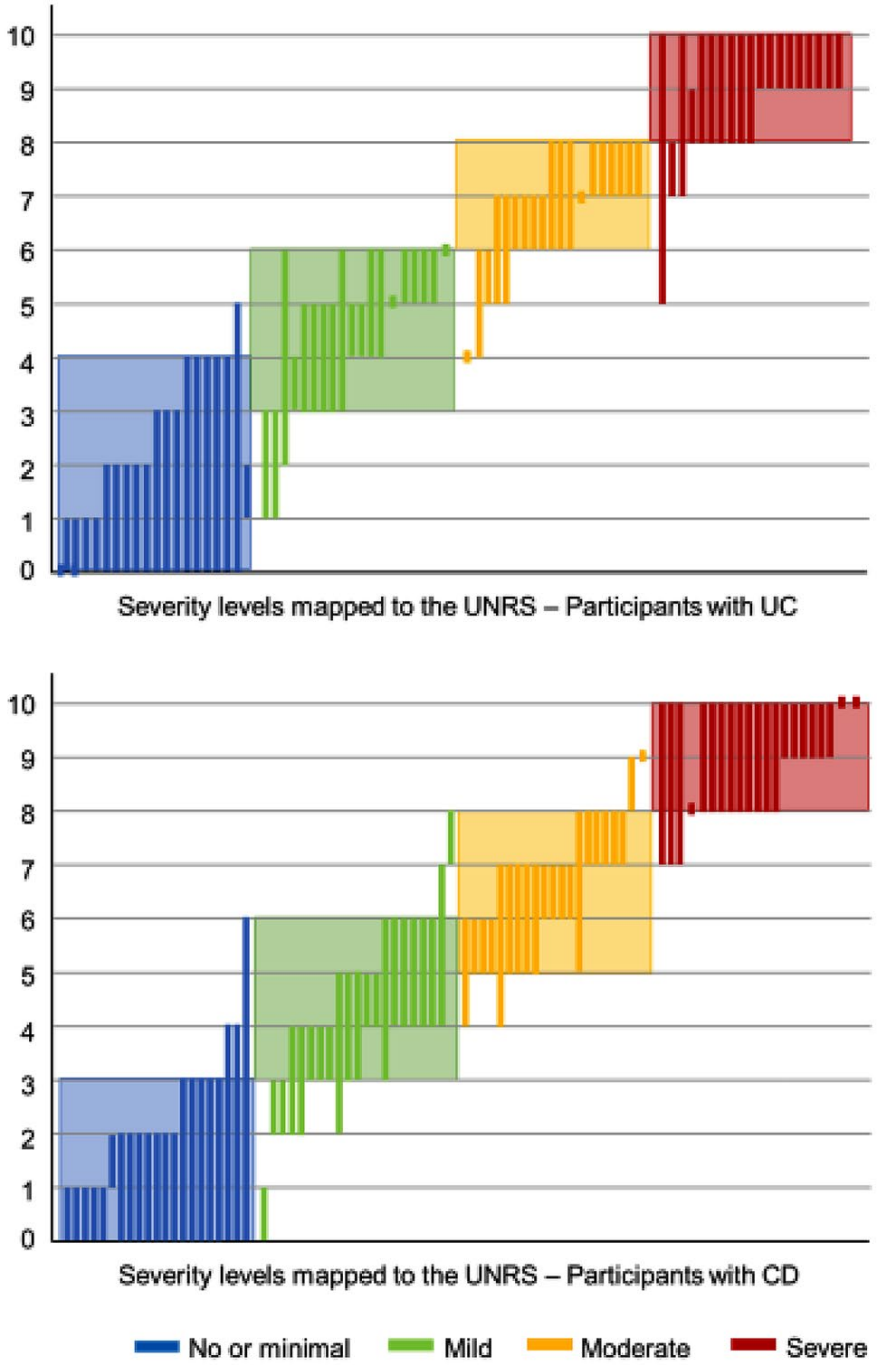
Severity levels mapped to the UNRS. Participants were asked “What would “no or minimal”/”mild range”/“moderate range”/“severe range” be on the 0–10 scale?” Shaded boxes represent the overall consensus for the ranges, and bars represent individual participant responses for each level. *CD* Crohn’s disease, *UNRS* Urgency Numeric Rating Scale, *UC* ulcerative colitis

Participants were asked to map the score ranges on the UNRS scale (0–10) that would correspond to “no or minimal,” “mild,” “moderate,” and “severe” bowel urgency severity (Fig. 2). Participants with UC selected scores from 0 to 5 on the UNRS for the “no or minimal” level, with broad consensus around 0–4. Ranges of 0–2 and 0–4 selected by five participants each. For “mild” bowel urgency, the consensus range was 3–6, with ranges of 3–5 and 5–6 selected by four participants each. For “moderate” bowel urgency, the consensus range was 6–8 and the most frequently selected ranges were 7–8 (*n* = 5), 6–7 (*n* = 4), and 6–8 (*n* = 3). For “severe” bowel urgency, the consensus range was 8–10 and the most frequently selected ranges were 9–10 (*n* = 9) and 8–10 (*n* = 6). Among participants with CD, scores selected for “no or minimal” bowel urgency were 0–6 on the UNRS, with broad consensus around 0–3. The most chosen ranges were 0–2 (*n* = 7), 0–3 (*n* = 5), and 0–1 (*n* = 5). Ranges selected to indicate “mild” bowel urgency were more variable; the consensus range was 5–8 and the most frequently chosen were 4–6 (*n* = 5) and 3–4 (*n* = 3). For the “severe” range there was broad consensus around 8–10, with nine participants choosing 8–10 and six choosing 9–10.

**Fig. 3:**
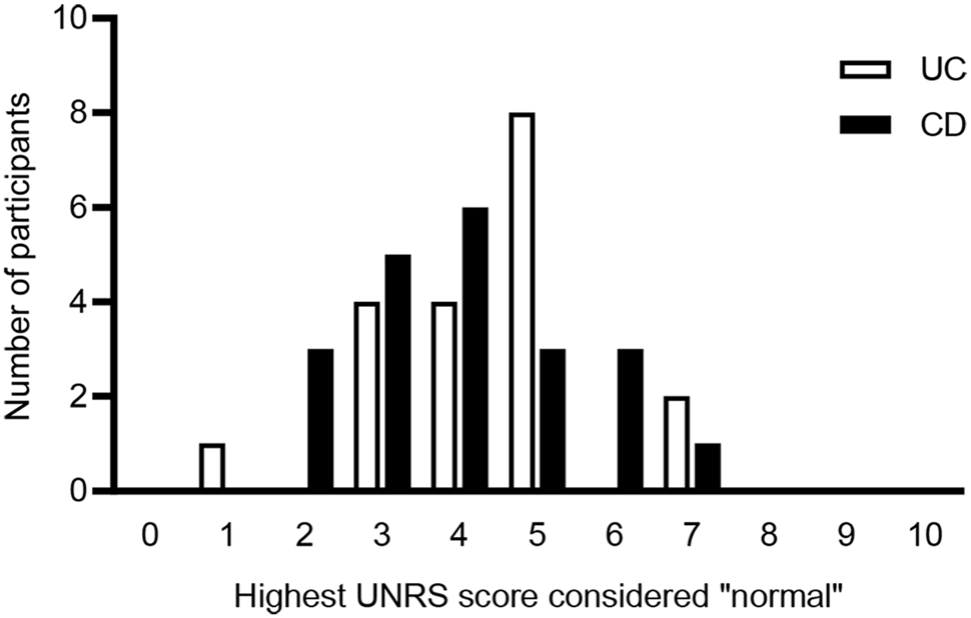
Highest number chosen on UNRS that could still be considered “normal” bowel urgency. Participants were asked “What is the highest number you could choose on this scale and still consider the amount of urgency you were having to be “normal,” “controlled,” or [in remission]?” *CD* Crohn’s disease, *UC* ulcerative colitis, *UNRS* Urgency Numeric Rating Scale

Participants were asked to indicate the highest score on the UNRS that they would still consider as “normal” or “in remission” (Fig. 3). For participants with UC, the highest proportion (*n* = 8) selected a 5, and the median score was 5 (range 1–7), and for participants with CD, the highest proportion selected a 4 (*n* = 6) or a 3 (*n* = 5), and the median score was 4 (range 2–7). When considering their answer, participants with UC reflected on their ability to make plans and function without excessive hurry or worry. Participants with CD also reported considering having the freedom to partake in daily activities without a sense of worry, as well as the ability to socialize more and being in control of when to have a bowel movement.

## Discussion

This study confirmed that, as seen in UC [10–12], bowel urgency is a common, long-term burden for patients with CD, affecting physical, social, emotional, and professional aspects of their lives. In both UC and CD, bowel urgency interferes with traveling, socializing, and going to public places or events; causes some patients to have to excessively plan; and results in patients feeling anxious, embarrassed, or frustrated. Bowel urgency also frequently affects work, causing difficulty completing tasks, absenteeism, and having to resign, retire early, or apply for disability.

When patients experience bowel urgency, they may have little warning before they need to have a bowel movement. As a result, they often need to organize activities around having access to a toilet to avoid an incident of fecal incontinence, something that almost all participants in this study experienced many times, and some even many times each week. This contrasts with a previous retrospective study in which only 14% of patients with inflammatory bowel disease reported fecal incontinence [21]. This difference is likely due to the current analysis being restricted to patients with moderate-to-severe disease who had experienced bowel urgency within the previous 3 months.

An important objective of this study was to uncover how patients with UC or CD describe bowel urgency remission. Most patients in this study considered remission of bowel urgency to be a state with a normal or reduced number of bowel movements and no or less frequent bowel urgency. To these participants, attaining some level of normalcy from bowel urgency remission would mean being able to partake in daily activities without a sense of worry, being able to socialize more, being in control of when to have a bowel movement, or being able to make plans and function without excessive hurry or worry.

Despite being common in patients with UC and CD, bowel urgency is typically underreported [22, 23], and healthcare practitioners may not adequately perceive its importance as a clinical symptom [8, 23]. Thus, patient-reported outcome measures for rating bowel urgency severity can be helpful when assessing treatment response and remission [8] and making decisions about treatment changes [24]. The UNRS is a single-item alternative to more complex tools for assessing patient-reported bowel urgency in moderate-to-severe UC and CD [25]. The current study confirmed that the UNRS score ranges adequately represent the severity of bowel urgency. It also found that most participants could even experience bowel urgency of moderate severity (a score of up to 5 [UC] or 4 [CD] on the UNRS) and still consider it to be “normal” or in remission. This contrasts with a previous psychometric evaluation that concluded that a UNRS score of ≤ 1 represented a threshold closely associated with clinical, endoscopic, and histologic remission [18].

A potential limitation of this study is that it included a relatively small patient sample, all US-based and mostly female, non-Hispanic, White, and in full-time employment. Also, although this study examined remission, it did not examine what would constitute a clinically meaningful improvement. A previous psychometric analysis found that a ≥ 3-point improvement on the UNRS represented a meaningful improvement in bowel urgency in patients with moderate-to-severe UC [18]. However, whether that threshold would also apply to patients with moderate-to-severe CD remains to be established.

## Conclusions

This study adds to our understanding of the symptom and severity of bowel urgency in patients with UC and CD, and it confirms that bowel urgency is also an important symptom to patients with CD that merits investigation in clinical trials. Finally, the study supports the UNRS as an appropriate tool to evaluate bowel urgency in clinical trials of moderate-to-severe UC or CD. Patients with moderate-to-severe UC or CD may consider bowel urgency to be in remission even at UNRS scores as high as 4 or 5.

## Data Availability

The datasets used and/or analyzed during the current study are available from the corresponding author on reasonable request.

## Abbreviations

CD: Crohn’s disease
SD: Standard deviation
UC: Ulcerative colitis
UNRS: Urgency Numeric Rating Scale
